# Profile and Areal Surface Parameters for Fatigue Fracture Characterisation

**DOI:** 10.3390/ma13173691

**Published:** 2020-08-20

**Authors:** Wojciech Macek, Ricardo Branco, Mirosław Szala, Zbigniew Marciniak, Robert Ulewicz, Norbert Sczygiol, Piotr Kardasz

**Affiliations:** 1 Opole University of Technology, 76 Proszkowska St., 45-758 Opole, Poland; 2 CEMMPRE, Department of Mechanical Engineering, University of Coimbra, Rua Luís Santos, 3030-788 Coimbra, Portugal; ricardo.branco@dem.uc.pt; 3 Department of Materials Engineering, Faculty of Mechanical Engineering, Lublin University of Technology, Nadbystrzycka 36D, 20-618 Lublin, Poland; m.szala@pollub.pl; 4 Department of Mechanics and Machine Design, Opole University of Technology, ul. Mikolajczyka 5, 45-271 Opole, Poland; z.marciniak@po.edu.pl; 5 Department of Production Engineering and Safety, Czestochowa University of Technology, 42-201 Czestochowa, Poland; robert.ulewicz@wz.pcz.pl; 6 Faculty of Mechanical Engineering and Computer Science, Czestochowa University of Technology, 42-201 Czestochowa, Poland; norbert.sczygiol@pcz.pl; 7 Wroclaw School of Information Technology, Lutra 4, 54-239 Wrocław, Poland; pkardasz@horyzont.eu

**Keywords:** surface metrology, surface topography, bending–torsion fatigue, fatigue fracture

## Abstract

Post-mortem characterisation is a pivotal tool to trace back to the origin of structural failures in modern engineering analyses. This work compared both the crack propagation and rupture roughness profiles based on areal parameters for total fracture area. Notched and smooth samples made of weather-resistant structural steel (10HNAP), popular S355J2 structural steel and aluminium alloy AW-2017A under bending, torsion and combined bending–torsion were investigated. After the fatigue tests, fatigue fractures were measured with an optical profilometer, and the relevant surface parameters were critically compared. The results showed a great impact of the loading scenario on both the local profiles and total fracture areas. Both approaches (local and total fracture zones) for specimens with different geometries were investigated. For all specimens, measured texture parameters decreased in the following order: total area, rupture area and propagation area.

## 1. Introduction

Post-mortem analysis is a fundamental engineering procedure to identify the damage accumulation mechanisms associated with fatigue failure. This analysis may provide important clues to improve a material’s performance, to evaluate both structural and mechanical properties and to mitigate the damage mechanisms [1,2,3].

Much research connected with surface metrology has been focused on extensive investigations in which 3D surface roughness parameters have been presented in light of the relationship between surface properties and operation properties [4,5,6,7,8]. Although this analysis provides useful information, post-failure fractographic surface examinations allow the cause of failure in materials to be determined [9,10,11,12]. Researchers have studied the characteristics of fractured surfaces using observational tools, from macro- to nanoscale [13,14,15,16,17]. Unfortunately, even though advanced methods such as optical coherence tomography [18], scanning acoustic microscopy [19] or energy response approach based on strain energy density histories during variable loading [20,21] are described in the literature, usually simple-fracture qualitative analysis conducted with scanning electron microscopy is employed for the evaluation of surface fractures resulting from impact [22,23], tensile strength [24,25], fatigue [5,19], ultra-high fatigue [26], adhesion testing [27] or even wear damage description [28,29]. However, few studies have investigated the use of profile and surface roughness as a tool for fatigue fracture characterisation.

Moreover, there are few papers devoted to the quantitative analysis of fracture surfaces in elements undergoing combined bending–torsion loading histories [30,31,32,33]. Therefore, the present study aimed to investigate bending–torsion fatigue fractures in order to establish the dependence between the loading scenario and the characteristic features of their surfaces. The literature describes the influence of loading conditions on the topography of fracture surfaces via either local terms (i.e., crack propagation and rupture) [34,35,36,37,38] or based on analysis of the total fracture area [39,40]. In this work, the results of both approaches (local and total fracture zones) for specimens with different geometries were compared for three materials. The local approach was defined as the application of linear measurement, and therefore the Ra parameter. Two sections, Ra propagation and Ra rupture, were measured in the study. The first was measured at a distance of about 1 mm from the crack initiation, while the second was measured close to the splitting of the sample into two parts. The total approach was defined by the Sa parameter. In this case, almost an entire fracture surface was analysed. For different loading scenarios (which encompassed bending, torsion and bending–torsion), 2D and 3D surface texture measurement methods were adopted, and the main results were critically compared.

## 2. Materials and Methods of Measurement

### 2.1. Materials and Specimens

The material grades studied in the present research were: (a) 10HNAP weather-resistant structural steel [39]; (b) S355J2 structural steel [41] and (c) AW-2017A-T4 aluminium alloy [42]. Components manufactured from the tested metal alloys are popular in the machine building industry, and therefore can undergo fatigue. The nominal chemical composition and mechanical properties are summarized in Table 1 and Table 2, respectively.

**Figure 1:**
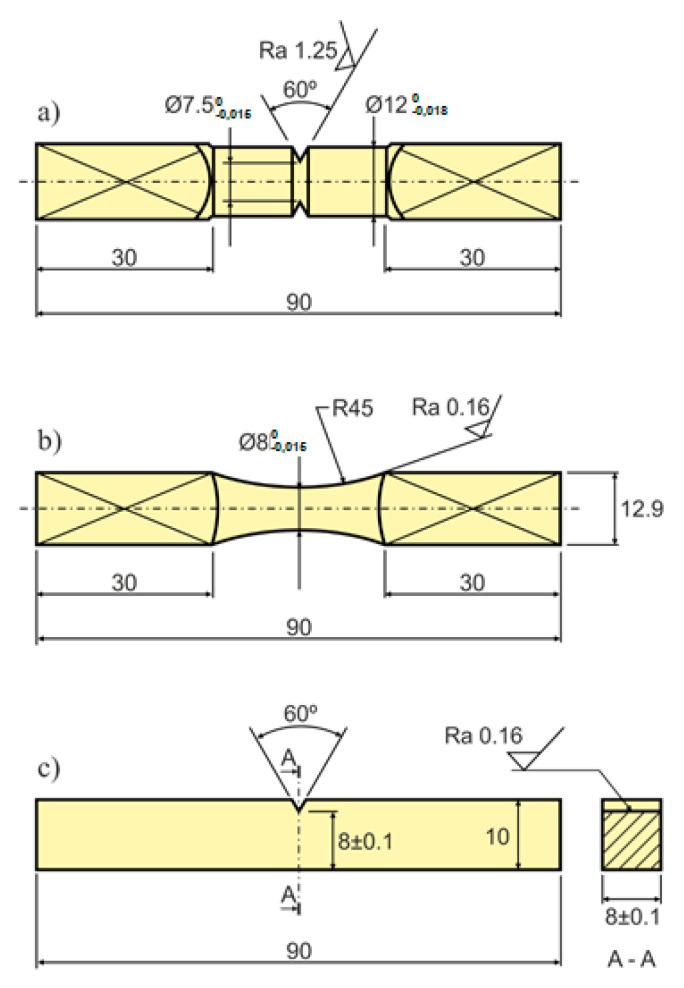
Specimen geometries: (**a**) V-notched circular cross section specimen made of 10HNAP steel; (**b**) smooth circular cross section specimen made of S355J2 steel; and (**c**) V-notched rectangular cross section specimen made of AW-2017A-T4 aluminium alloy (dimensions in millimetres).

Specimens used in the experimental fatigue experiment are depicted in Figure 1, and comprised: (a) cylindrical 10HNAP steel specimens with a circumferential V-notch (Figure 1a); specimens with a circular cross section (Figure 1b) and AW-2017A-T4 aluminium rectangular cross section specimens with a V-notch (Figure 1c). Both V-shaped configurations had external, unilateral, sharp and blunt one-sided notches, with radii r = 0.2 mm, 5 mm, 10 mm and 22.5 mm and notch angles of 60°.

### 2.2. Loading Histories

**Figure 2:**
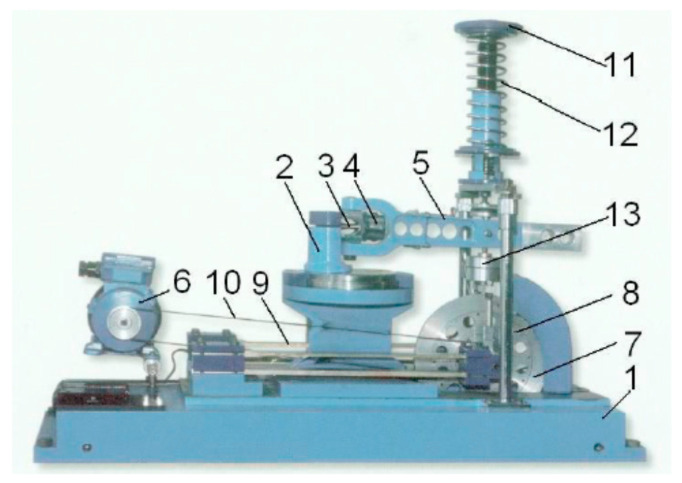
MZGS-100 fatigue test stand where: 1—bed, 2—rotational head with a holder, 3—specimen, 4—holder, 5—lever (effective length = 0.2 m), 6—motor, 7—rotating disk, 8—unbalanced mass, 9—flat springs, 10—driving belt, 11—spring actuator, 12—spring, 13—hydraulic connector.

**Figure 3:**
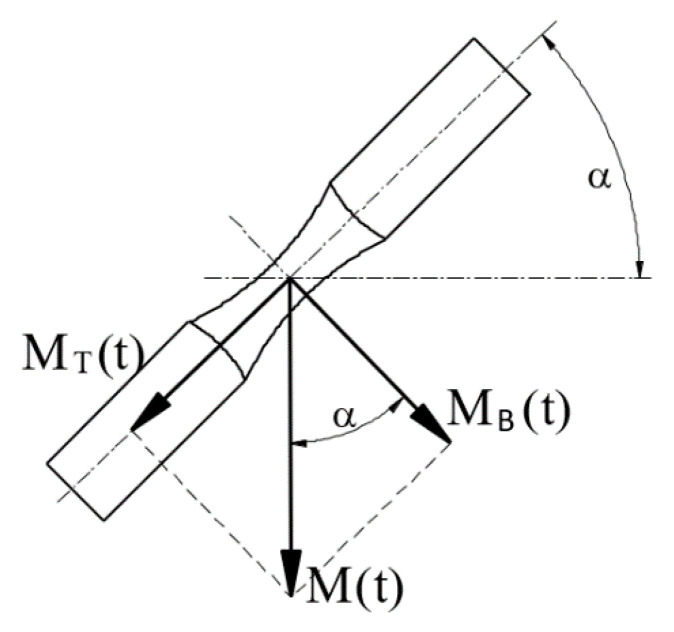
Configuration of specimen loading where: M_T_—torsion, M_B_—bending, M—total moment.

All specimen geometries were tested under combined bending–torsion, bending and torsion (except geometry b). The fatigue test of 10HNAP steel samples was tested by Achtelik [44]. Stationary and ergodic random loadings had a normal probability distribution and wide-band frequency spectra from 0 to 60 Hz. In the case of mixed loading, nominal normal stress amplitudes were equal to nominal shear stress amplitudes (i.e., σ_a_ = τ_a_). Fatigue tests of S355J2 steel specimens carried out by Marciniak [41] encompassed non-proportional bending–torsion histories with different ratios of the maximum shear stress to the maximum normal stress (i.e., λ = τ_max_/σ_max_). In the case of the AW-2017A-T4 aluminium alloy specimens, tests were conducted by Faszynka [42] under different bending-to-torsion amplitude ratios. The stress ratios (R) used in this experimental campaign were R = −1, −0.5 and 0 (Table 3) [32]. All tests were carried out using the MZGS fatigue test stand type machine. However, loads were caused by different methods. The main idea is presented in Figure 2, where the unbalanced disk (7) during rotations caused vibrations on the flat spring (9) and then transmitted them through the rods and the lever (5) on a specimen (3). Figure 3 presents the configuration of the specimen loads. Other tests and fatigue stands are described in [41,43].

### 2.3. Surface Parameter Measurement and Calculation

**Figure 4:**
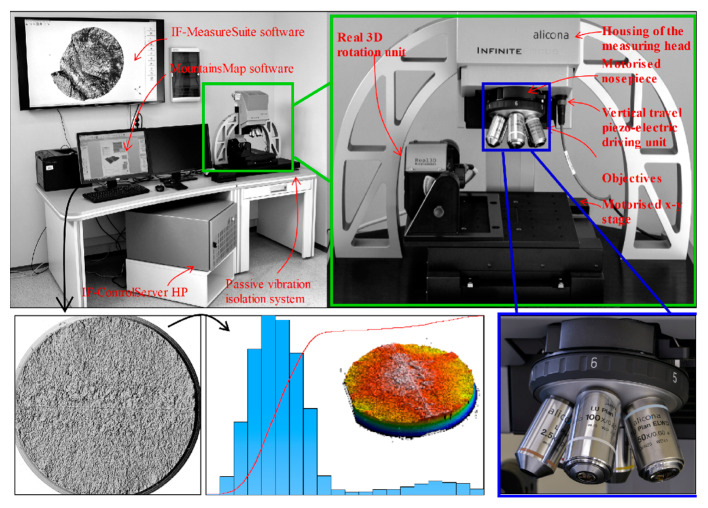
InfiniteFocus IF G4 measurement device used in surface metrology and The Abbott-Firestone curves (middle bottom picture).

**Figure 5:**
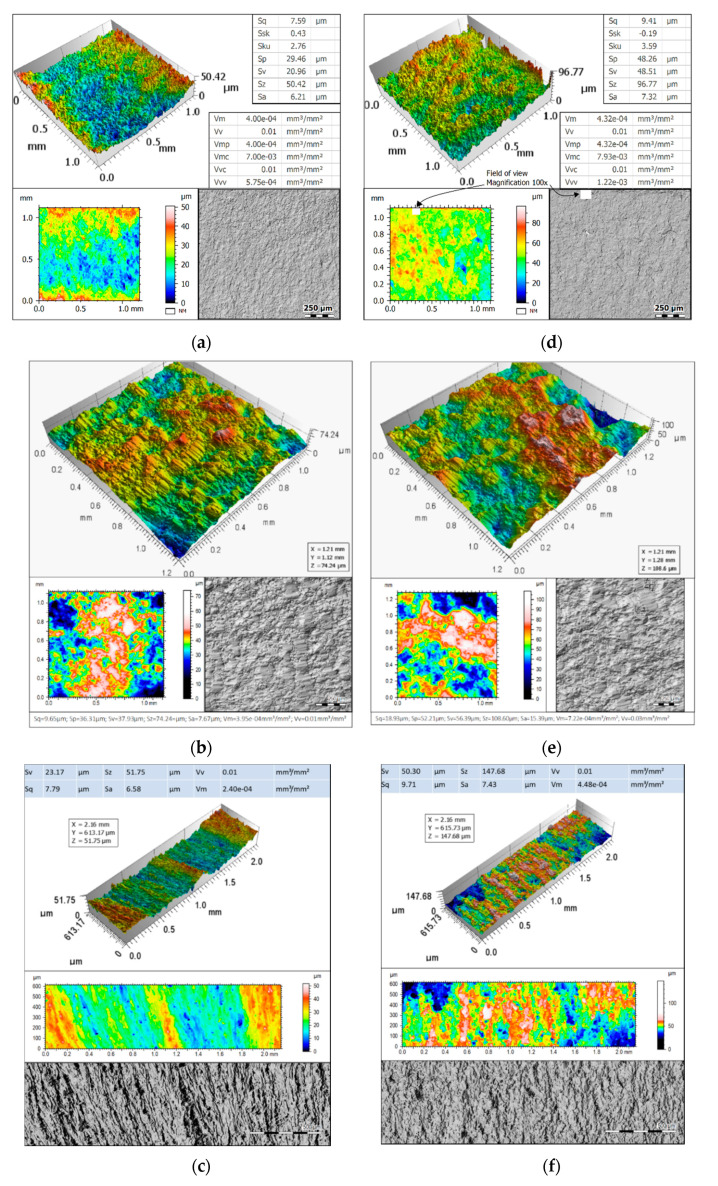
Propagation area view of the specimen made of: (**a**) 10HNAP steel; (**b**) S355J2 steel; and (**c**) AW-2017A-T4 aluminium alloy. Rupture area view of the specimen made of: (**d**) 10HNAP steel; (**e**) S355J2, steel; and (**f**) AW-2017A-T4 aluminium alloy.

Fracture surface analysis was performed by Macek [34,35,39] using an optical 3D test stand that facilitated the acquisition of data sets at a high depth of focus [45,46]. The failed specimens were observed under 10× magnification using an Alicona G4 InfiniteFocus (Alicona Imaging GmbH, Graz, Austria) as described previously [47]. Due to the restricted field of view, nine rows by seven columns were stitched together to map the entire fracture area. Each individual micrograph had a vertical resolution of 79.3 nm with a lateral resolution of 3.91 µm. The abovementioned measurement device, exhibited in the upper part of Figure 4, was operated via IF-MeasureSuite software (version 5.1, Alicona Imaging GmbH, Graz, Austria), while the measurement of surface features was conducted using MountainsMap software (version 7.4, Digital Surf, Besançon, France). Alicona (*.al3d) files were imported into the surface metrology software MountainsMap and resampled into height maps at a resolution automatically determined by the software. Surfaces were analysed in relative coordinates (X, Y, and Z axes) with the Z axis in heights from the lowest point by default. No additional filters were used. Fatigue fracture surfaces were measured for local (propagation and rupture) profiles and for total areas. Figure 5 shows examples of the propagation areas and main surface parameters as well as rupture areas and main texture parameters observed in the experiments for the three metal alloys studied.

To check the fracture surface dependency on the fatigue loading history, selected parameters, reported among others in Figure 5, were measured and calculated. Table 4 defines the used parameters according to the ISO 25178 standard.

It is known that the microrelief of fatigue fracture surface is determined by the material properties and the stress intensity factor in the tip of the initial crack; therefore, the parameters of the microrelief depend on the stress amplitude and the fatigue crack length. When testing ductile materials, the height of the fracture profile usually increases with increasing crack length, and at the stage of the fatigue crack propagation, three zones with different roughness are found: the initial zone with a predominant shear microrelief; the zone with striation microrelief; and the zone of accelerated crack growth, in which striations and dimples are observed. With an increase in the stress amplitude, the size of the zones changes, the zone with striations decreases and the zone of rupture grows. When testing brittle or quasi-brittle materials, the height of the fracture profile often decreases with increasing crack length as a result of the formation of facets of cleavage or intergranular fracture [48,49,50,51]. Overall, there were obvious differences in topography for propagation or rupture, particularly the coarser areas. Ra (Equation (1)) averages all peaks and valleys of the roughness profile and then neutralizes the few outlying points, so that the extreme points have no significant impact on the final results. Sa, as expressed in Equation (2), represents the mean height of the surface, according to the ISO 25178 standard. Their functionality was analysed later in the study.

The Abbott–Firestone curves (see centre of Figure 4) provide important information on the surface properties in a systematic and quantitative approach. In the example chart, the Abbott–Firestone curve shows the cumulative height distribution histogram. The horizontal axis represents the measured scale in depth of the surface, and the vertical axis depicts percentage of the whole population of data. The shape of the curve is distilled into several of the surface roughness parameters [52]. The distributions of the surface highlights that the crack initiation region had a smoother surface without asperities.

(1) $$Ra\;=\;\frac{1}{lr}\int_{0}^{lr}\left|z\left(x\right)\right|dx$$

(2) $$Sa\;=\;\frac{1}{A}\underset{A}{\iint }\left|z\left(x,y\right)\right|dxdy$$

## 3. Results and Discussion

**Figure 6:**
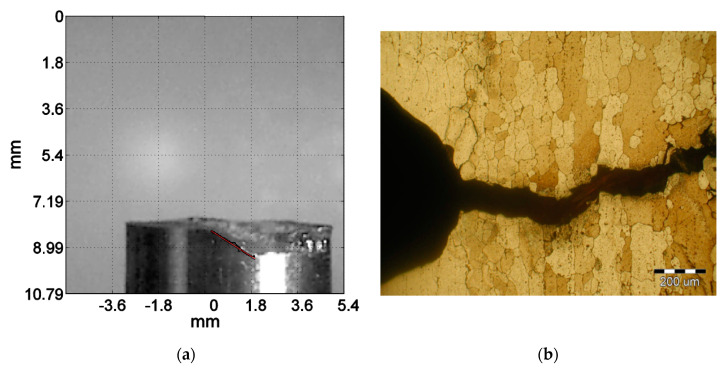
(**a**) Side view of S355J2 sample (lines show the place of initiation); (**b**) AW-2017A-T4 aluminium alloy.

The fracture plane analysis revealed that, in S355J2 steel samples, the cracks initiated in the plane of maximum shear stresses and then propagated in the plane of maximum normal stresses (Figure 6a). In contrast, in notched samples, due to the significant predominance of normal stresses, initiation and propagation of cracks took place in the plane of maximum normal stresses (Figure 6b).

**Figure 7:**
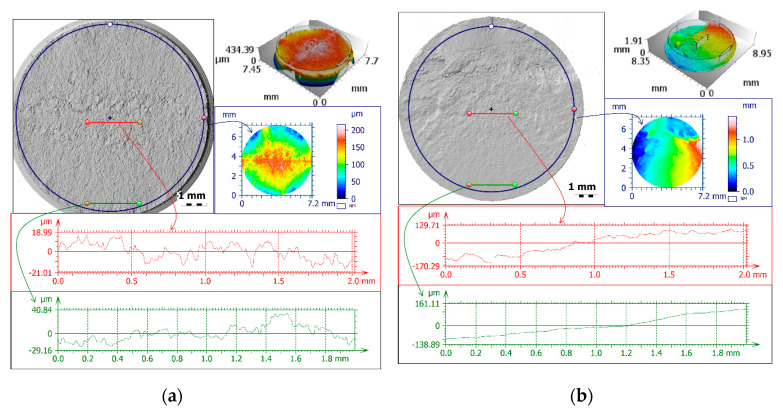

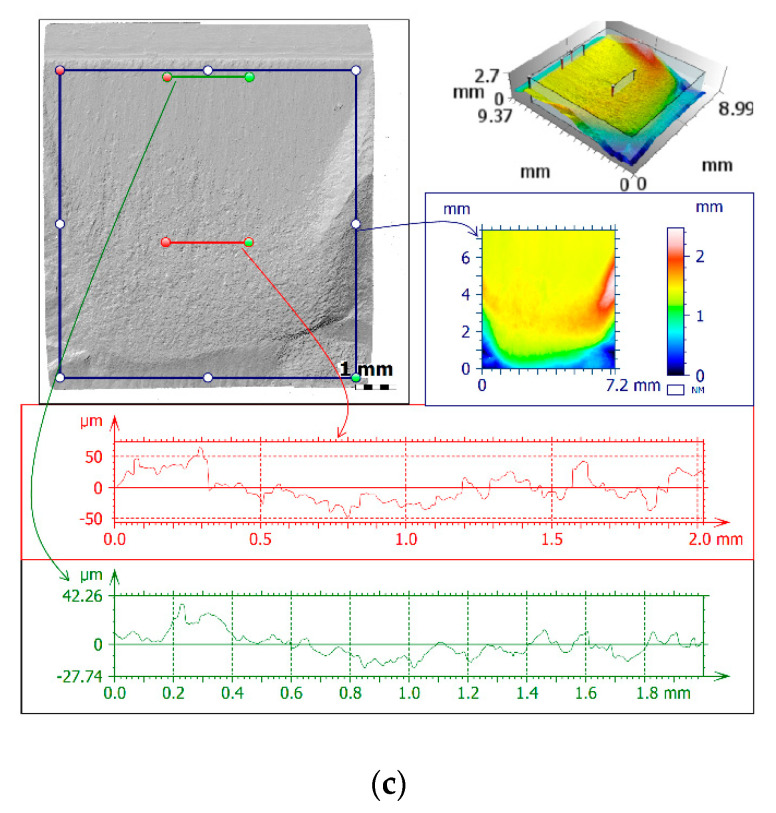
Fractures with marked crack propagation profile (green), rupture roughness profile (red), and extracted total area for exemplary specimens made of: (**a**) 10HNAP steel; (**b**) S355J2 steel; and (**c**) AW-2017A-T4 aluminium alloy.

Figure 7 shows representative surface texture measurement results for both total areas and for local profiles (in terms of propagation and rupture). In Figure 7, the blue frames represent total fracture areas, the propagation profile is shown in green, and the rupture profile is shown in red. In contrast, in the black frame and without the frame, the whole views of fatigue fractures are presented with the measurement areas and profiles marked. In fact, as anticipated in Figure 5, comparison of the different evaluated parameters for the three materials showed important differences, like greater roughness—for both areas and profiles—in the rupture zone than in the propagation zone.

Thanks to the 3D parameters (S-), surface shape and direction can be assessed. The fracture surface presented large disorderly peaks. Therefore, in the case of the 2D parameters (R-), the randomness and the fortuitousness of the measurement profile direction and location on the surface are important.

Some generic features of the tested specimens (e.g., the dependence of roughness parameters in individual areas on the bending, torsion or combined loading type) that were initially noticed in the measurement results were analysed in this part of the study. For further analysis, Ra and Sa were selected from R- and S-parameters. These parameters demonstrated the best fit, evident dependence on the loading condition and widespread use, regardless of the measuring technique, which was also presented for other studies of fracture surface topography (e.g., in [8]).

**Figure 8:**
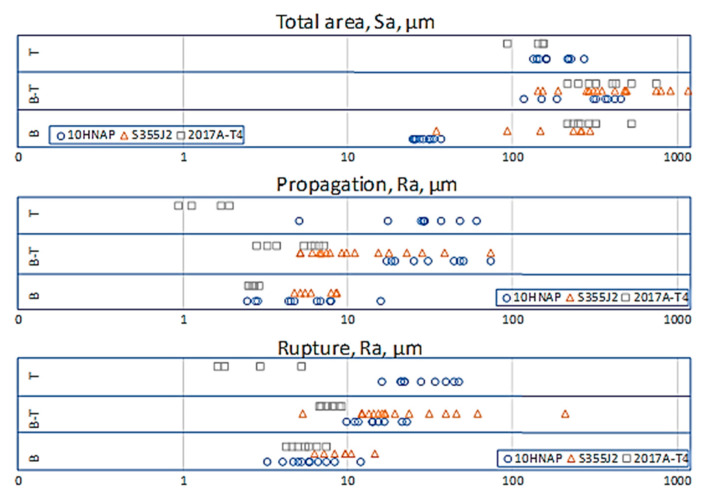
Results for Ra and Sa by type of loading.

Figure 8 presents an extract of all analysed results for Ra and Sa, by type of loading. Without qualifying the method and place of measuring the fractured surface, it can be seen that, generally, the highest values occurred for a mixed-mode loading and the lowest for torsion.

**Figure 9:**
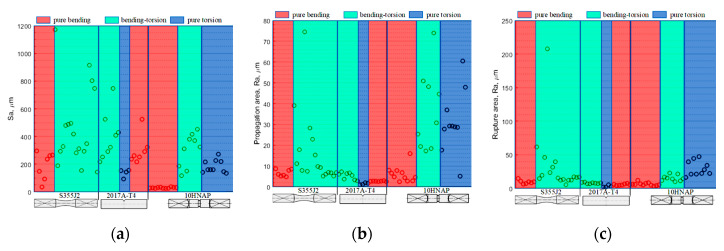
Surface parameters for the investigated fracture areas: (**a**) total area; (**b**) propagation profile; and (**c**) rupture profile.

Figure 9a–c presents the results of all specimens divided into three analysed ways of identifying the fracture (i.e., total area, propagation profile and rupture profile, respectively). For the results of measurements of the total fracture surface Sa parameter (likewise Ra for the propagation profile), the highest values and the greatest dispersion were obtained for bending–torsion. The 10HNAP specimens were the exception. For this case, the torsion Ra parameter had the highest values (Table 5). In the case of torsion, for which only measurements of samples (a) and (c) were taken, the latter took the lowest values.

**Figure 10:**
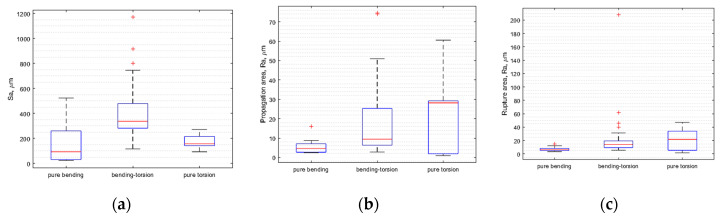
Box plots for results: (**a**) total fracture area; (**b**) propagation profile; (**c**) rupture profile.

In statistical terms, the dependence of surface parameters on the type of loading was presented using box plots with, among other things, percentiles. On each box (Figure 10), the central mark indicates the median, and the bottom and top edges of the box indicate the 25th and 75th percentiles, respectively. The whiskers extend to the most extreme data points not considered outliers, and the outliers are plotted individually using the “+” symbol.

As shown in Figure 10a, the median Sa for all bending specimens was approximately 100 μm. The minimum value was about 10 μm, and the maximum value was about 550 μm. For bending–torsion and torsion, respectively, these values were median Sa 340 μm and 150 μm, minimum values 110 μm and 100 μm, and maximum values 750 μm and 270 μm. Next, taking into account only the medians for propagation area (Figure 10b), Ra was about 5 μm for bending, 10 μm for bending–torsion and 28 μm for torsion loadings. For rupture area, the measurements were 7 μm, 14 μm and 21 μm. Table 6 summarises the medians extremum of measurement results for all specimens broken down into reference area and loading scenario. Clear relationships between the size of surface parameters for individual analysed fracture zones are shown in Figure 4, Figure 5, Figure 6, Figure 7, Figure 8, Figure 9 and Figure 10.

The value of the Ra parameter depends on the place and direction of orientation of the measuring section, and the values for individual measured zones differ. For example, the influence of the asymmetry factor is ambiguous because, for bending, the Ra values increase with the stress ratio R, and for the combination of bending with torsion, this relationship is reversed. Therefore, the results may be ambiguous. However, the influence of the stress ratio R on the value of the Sa parameter showed an increase of this parameter along with an increase in the cycle asymmetry factor from −1 to 0, which is consistent with previously obtained results [53]. The magnitude of the load also affected all roughness parameters. The values of the roughness parameters Sa and Ra changed with the load; however, this relationship did not change proportionally. For example, for the aluminium alloy, a double increase in the load for the coefficient R = 0 caused the Sa parameter to increase by 10%, while the Ra propagation parameters decreased by 14% and Ra rupture by 25%. In the case of the tested steels, when analysing the influence of load on roughness, one should also pay attention to the influence of the ratio of shear stresses to normal stresses. When the samples were subjected to loads with a predominance of bending stresses, the parameters Sa and Ra decreased, while if the influence of shear stresses increased, the roughness parameters increased. Similar conclusions - linear functions between topography parameters and fatigue variables were found by authors in [54]. It is worth noting that the notch, which was the initiator of fatigue cracks and which increased the effect of normal stresses, had a significant influence on the parameter values. Among the examined materials, the smallest differences in the variability of Ra parameters were presented by the aluminium alloy, while in the case of steel, the spans were greater. The Sa parameter, as the surface parameter, is independent of the orientation of the measurement direction, making it a more universal value.

## 4. Conclusions

This study presents methods for the metrological characterisation and comparison of fatigued fractures using post-failure measurements. Combining various techniques and fields of science ensures a more complete analysis of the issue. The investigation of the entire fracture surface taking various factors into account gives greater opportunities to find the causes of failures. The proposed methodologies were tested under different loading histories, namely, bending, torsion and combined bending–torsion; in different materials (structural steels and aluminium alloy); and with different specimen geometries (smooth and notched samples). Internal microstructural defects can be related to the formation of crack initiation and propagation, influencing the surface shaping and therefore the roughness parameters. However, based on our analyses and comparisons, the following general conclusions can be drawn:

- The entire total area method is more universal and burdened with less error than the subjective method of measurement in individual fracture zones for various shape-types of fatigue-tested samples.
- This method is suitable for assessment, and supplements the testing of materials damaged as a result of fatigue loads, for various shapes of the tested detail.
- Both profile and areal surface parameters are essential for fatigue fracture mechanism characterisation. However, if we compare profiles of R-roughness and areal S-roughness parameters, the latter give complete information, because a single line cannot identify pits or valleys, and shows the relationship between surface functions. 3D measurements give far more comprehensive information than 2D profiles or sections.
- For both zones (propagation and rupture), the median Ra increases along with the increase in the proportion of torsional loadings. Otherwise, for total area, the mixed mode causes the highest average Sa value, and this conclusion can be considered appropriate given the greater accuracy of this method. This is also confirmed by the results for the individual types of specimens.
- The Sa parameter is more universal, independent of measurement orientation.

Further research should focus on the suitability of the entire total area method for a wider range of metallic and composite materials, and types of loadings.

## Figures and Tables

**Table 1:** Chemical composition of the tested alloys (wt.%) [41,42,43,44].

Element	10HNAP	S355J2	AW-2017A-T4
C	0.115	0.21	-
Si	0.41	0.42	0.45
Mn	0.71	1.46	0.65
P	0.082	0.019	-
S	0.028	0.046	-
Cr	0.81	0.09	0.10
Ni	0.50	0.04	-
Cu	0.30	0.17	4.15
Zn	-	-	0.50
Mg	-	-	0.69
Ti	-	-	0.20
Al	-	-	Balance
Fe	Balance	Balance	0.70

**Table 2:** Main mechanical properties of the tested alloys.

Material Properties	10HNAP [44]	S355J2 [41]	AW-2017A-T4 [42]
Ultimate tensile stress, σ_u_ (MPa)	566	533	480
Yield stress, σ_y_ (MPa)	418	358	382
Elongation A_10_ (%)	A_10_ = 30.1	A_5_ = 21	A_5_ = 15
Reduction of area RA (%)	36.5	50	50
Young’s modulus E (GPa)	215	210	72
Poisson’s ratio ν	0.29	0.30	0.32

**Table 3:** The loading parameters.

Material	Specimen		
	λ	R	Ref.
10HNAP	0; 0.5; ∞	−1	[44]
S355J2	0; 0.16−0.6	−1	[41]
AW-2017A	0; 0.18−0.44; ∞	−1; −0.5; 0; 1	[35,42]

**Table 4:** Selected parameters for the fatigue fracture surface description according to ISO 25178.

Height Parameters			
Sq	µm	Root-mean-square height	$\mathrm{Sq}\;=\sqrt{\frac{1}{A}\iint_{A}z^{2}\left(x,y\right)\mathrm{dxdy}}$
Ssk	-	Skewness	$\;\mathrm{Ssk}\;=\frac{1}{{\mathrm{Sq}}^{3}}(\frac{1}{A}\underset{A}{\iint }z^{3}\left(x,y\right)\mathrm{dxdy})$
Sku	-	Kurtosis	$\;\mathrm{Sku}\;=\frac{1}{{\mathrm{Sq}}^{4}}(\frac{1}{A}\underset{A}{\iint }z^{4}\left(x,y\right)\mathrm{dxdy})$
Sp	µm	Maximum peak height	Sp = Sz − Sv
Sv	µm	Maximum pit height	Absolute value of the height of the largest pit within the defined area
Sz	µm	Maximum height	Height between the highest peak and the deepest valley
Sa	µm	Arithmetical mean height	Mean surface roughness $\mathrm{SaM}=M\frac{1}{A}\iint_{A}\left|z\left(x,y\right)\right|\mathrm{dxdy}$
**Functional Parameters (Volume)**			
Vm	mm^3^/mm^2^	Material volume	Parameters describing the characteristics of the volume of the appropriate size to the surface area of the surface being examined
Vv	mm^3^/mm^2^	Void volume	
Vmp	mm^3^/mm^2^	Peak material volume	
Vmc	mm^3^/mm^2^	Core material volume	
Vvc	mm^3^/mm^2^	Core void volume	
Vvv	mm^3^/mm^2^	Pit void volume	

**Table 5:** List of maximum and minimum values of surface parameters for individual reference area.

Method	Specimen		
	10HNAP	S355J2	AW-2017A
Total area max.	BT	BT	BT
Propagation profile max.	BT	BT	BT
Rupture profile max.	T	BT	BT
Total area min.	B	B	T
Propagation profile min.	B	B	T
Rupture profile min.	B	B	T

BT: bending–torsion; T: torsion; B: bending.

**Table 6:** Median extremum for individual reference areas.

Reference Area/Median Extremum	Bending	Bending–Torsion	Torsion
Total area	MIN.	MAX.	-
Propagation profile	MIN.	-	MAX.
Rupture profile	MIN.	-	MAX.

## References

[1] Ulewicz R., Nový F., Novák P., Palček P. (2019). The investigation of the fatigue failure of passenger carriage draw-hook. Eng. Fail. Anal..

[2] Branco R., Antunes F.V., Costa J.D., Yang F.P., Kuang Z.B. (2012). Determination of the Paris law constants in round bars from beach marks on fracture surfaces. Eng. Fract. Mech..

[3] Su Y., Yu F.-H., Han Q.-N., Shang Y.-B., Rui S.-S., Li J., Shi H.-J., Niu L.-S. (2019). Failure analysis of runway centerline light and effect of microstructure on mechanical properties. Eng. Fail. Anal..

[4] Branco R., Costa J.D., Berto F., Antunes F.V. (2017). Effect of loading orientation on fatigue behaviour in severely notched round bars under non-zero mean stress bending-torsion. Theor. Appl. Fract. Mech..

[5] Lesiuk G., Rymsza B., Rabiega J., Correia J.A.F.O., De Jesus A.M.P., Calcada R. (2019). Influence of loading direction on the static and fatigue fracture properties of the long term operated metallic materials. Eng. Fail. Anal..

[6] Azevedo C.R.F., Marques E.R. (2010). Three-dimensional analysis of fracture, corrosion and wear surfaces. Eng. Fail. Anal..

[7] Szala M., Walczak M., Pasierbiewicz K., Kamiński M. (2019). Cavitation erosion and sliding wear mechanisms of AlTiN and TiAlN films deposited on stainless steel substrate. Coatings.

[8] Macek W., Owsiński R., Trembacz J., Branco R. (2020). Three-dimensional fractographic analysis of total fracture areas in 6082 aluminium alloy specimens under fatigue bending with controlled damage degree. Mech. Mater..

[9] Becker W.T., Lampman S., Becker W.T., Shipley R.J. (2002). Fracture appearance and mechanisms of deformation and fracture. Failure Analysis and Prevention.

[10] Lynch S.P., Moutsos S. (2006). A brief history of fractography. J. Fail. Anal. Prev..

[11] Miletić I., Ilić A., Nikolić R.R., Ulewicz R., Ivanović L., Sczygiol N. (2020). Analysis of selected properties of Welded joints of the HSLA steels. Mater. Basel Switz..

[12] Taira S., Tanaka K., Ryu J.G. (1974). X-ray diffraction approach to the mechanics of fatigue and fracture in metals. Mech. Res. Commun..

[13] Leer B.V., Genc A., Passey R. (2017). Ga+ and Xe+ FIB milling and measurement of FIB damage in aluminum. Microsc. Microanal..

[14] Karbalaei Akbari M., Baharvandi H.R., Shirvanimoghaddam K. (2015). Tensile and fracture behavior of nano/micro TiB2 particle reinforced casting A356 aluminum alloy composites. Mater. Des..

[15] Lemaitre J. (1984). How to use damage mechanics. Nucl. Eng. Des..

[16] Kelly J., Mohammadi M. (2018). Uniaxial tensile behavior of sheet molded composite car hoods with different fibre contents under quasi-static strain rates. Mech. Res. Commun..

[17] Szala M., Łukasik D. (2018). Pitting corrosion of the resistance welding joints of stainless steel ventilation grille operated in swimming pool environment. Int. J. Corros..

[18] Hutiu G., Duma V.-F., Demian D., Bradu A., Podoleanu A.G. (2018). Assessment of ductile, brittle, and fatigue fractures of metals using optical coherence tomography. Metals.

[19] Kubit A., Trzepiecinski T., Faes K., Drabczyk M., Bochnowski W., Korzeniowski M. (2019). Analysis of the effect of structural defects on the fatigue strength of RFSSW joints using C-scan scanning acoustic microscopy and SEM. Fatigue Fract. Eng. Mater. Struct..

[20] Lesiuk G., Szata M., Rozumek D., Marciniak Z., Correia J., De Jesus A. (2018). Energy response of S355 and 41Cr4 steel during fatigue crack growth process. J. Strain Anal. Eng. Des..

[21] Kasprzyczak L., Macha E., Marciniak Z. Energy Parameter Control System of Strength Machine for Material Tests under Cyclic Bending and Torsion.

[22] Kowal M., Szala M. (2020). Diagnosis of the microstructural and mechanical properties of over century-old steel railway bridge components. Eng. Fail. Anal..

[23] Krawczyk J., Pacyna J., Bała P. (2015). Fracture toughness of steels with nickel content in respect of carbide morphology. Mater. Sci. Technol..

[24] Derpeński Ł. (2019). Ductile fracture behavior of notched aluminum alloy specimens under complex non-proportional load. Materials.

[25] Żebrowski R., Walczak M., Korga A., Iwan M., Szala M. (2019). Effect of shot peening on the mechanical properties and cytotoxicity behaviour of titanium implants produced by 3D printing technology. J. Healthc. Eng..

[26] Nový F., Bokůvka O., Trško L., Jambor M. (2019). Safe choice of structural steels in a region of ultra-high number of load cycles. Prod. Eng. Arch..

[27] Walczak M., Pasierbiewicz K., Szala M. (2019). Adhesion and mechanical properties of TiAlN and AlTiN magnetron sputtered coatings deposited on the DMSL titanium alloy substrate. Acta Phys. Pol. A.

[28] Maslarevic A., Bakic G.M., Djukic M.B., Rajicic B., Maksimovic V., Pavkov V. (2020). Microstructure and wear behavior of MMC coatings deposited by plasma transferred Arc welding and thermal flame spraying processes. Trans. Indian Inst. Met..

[29] Szala M., Dudek A., Maruszczyk A., Walczak M., Chmiel J., Kowal M. (2019). Effect of atmospheric plasma sprayed TiO2-10% NiAl cermet coating thickness on cavitation erosion, sliding and abrasive wear resistance. Acta Phys. Pol. A.

[30] Slámečka K., Pokluda J., Ponížil P., Major Š., Šandera P. (2008). On the topography of fracture surfaces in bending–torsion fatigue. Eng. Fract. Mech..

[31] de Freitas M., Reis L., Meggiolaro M.A., de Castro J.T.P. (2017). Stress scale factor and critical plane models under multiaxial proportional loading histories. Eng. Fract. Mech..

[32] Karolczuk A. (2006). Plastic strains and the macroscopic critical plane orientations under combined bending and torsion with constant and variable amplitudes. Eng. Fract. Mech..

[33] Santus C., Taylor D., Benedetti M. (2018). Experimental determination and sensitivity analysis of the fatigue critical distance obtained with rounded V-notched specimens. Int. J. Fatigue.

[34] Macek W., Wołczański T. (2017). Analysis of fracture roughness parameters of S355J2 steel and EN AW-2017A-T4 aluminium alloy. ITM Web Conf..

[35] Macek W., Faszynka S., Deptuła A., Świder J., Kciuk S., Trojnacki M. (2019). Fracture surface analysis of the EN AW-2017A-T4 specimens with rectangular section. Proceedings of the Mechatronics 2017—Ideas for Industrial Applications, Wisła, Poland, 2017.

[36] Singh A.K., Datta S., Chattopadhyay A., Riddick J.C., Hall A.J. (2019). Fatigue crack initiation and propagation behavior in Al-7075 alloy under in-phase bending-torsion loading. Int. J. Fatigue.

[37] Valoroso N., Debruyne G., Laverne J. (2014). A cohesive zone model with rate-sensitivity for fast crack propagation. Mech. Res. Commun..

[38] Martins R.F., Ferreira L., Reis L., Chambel P. (2016). Fatigue crack growth under cyclic torsional loading. Theor. Appl. Fract. Mech..

[39] Macek W. (2019). Post-failure fracture surface analysis of notched steel specimens after bending-torsion fatigue. Eng. Fail. Anal..

[40] Macek W. (2019). Fractal analysis of the bending-torsion fatigue fracture of aluminium alloy. Eng. Fail. Anal..

[41] Marciniak Z., Rozumek D., Macha E. (2008). Fatigue lives of 18G2A and 10HNAP steels under variable amplitude and random non-proportional bending with torsion loading. Int. J. Fatigue.

[42] Rozumek D., Faszynka S. (2017). Fatigue crack growth in 2017A-T4 alloy subjected to proportional bending with torsion. Frat. Ed Integrità Strutt..

[43] Rozumek D., Marciniak Z. (2008). Control system of the fatigue stand for material tests under combined bending with torsion loading and experimental results. Mech. Syst. Signal Process..

[44] Achtelik H., Lachowicz C., Lagoda T., Macha E. Fatigue characteristics of the notched specimens of 10HNAP steel under cyclic and random synchronous bending with torsion. Proceedings of the 2nd Annual Fatigue Meeting of Copernicus Contract CIPA CT940194.

[45] Kapłonek W., Nadolny K., Królczyk G.M. (2016). The use of focus-variation microscopy for the assessment of active surfaces of a new generation of coated abrasive tools. Meas. Sci. Rev..

[46] Newton L., Senin N., Gomez C., Danzl R., Helmli F., Blunt L., Leach R. (2019). Areal topography measurement of metal additive surfaces using focus variation microscopy. Addit. Manuf..

[47] Macek W., Rozumek D., Królczyk G.M. (2020). Surface topography analysis based on fatigue fractures obtained with bending of the 2017A-T4 alloy. Measurement.

[48] Neimitz A., Galkiewicz J., Lipiec S., Dzioba I. (2018). Estimation of the onset of crack growth in ductile materials. Materials.

[49] Kim J., Kang J.W., Lee D.-E., Kim D.Y. (2019). Methodology for evaluation of residual stress effect on small corner-crack initiation and growth. Materials.

[50] Djoković J.M., Nikolić R.R., Ulewicz R., Hadzima B. (2020). Interface crack approaching a three-material joint. Appl. Sci..

[51] Nikolić R.R., Djoković J.M., Hadzima B., Ulewicz R. (2020). Spot-weld service life estimate based on application of the interfacial crack concept. Materials.

[52] Stachowiak G.W., Batchelor A.W. (2005). Engineering Tribology.

[53] Deptuła A., Macek W., Partyka M.A. Analysis of loading history influence on fatigue and fracture surface parameters using the method of induction trees. Proceedings of the MATEC Web of Conferences.

[54] Macek W., Branco R., Trembacz J., Costa J.D., Ferreira J.A.M., Capela C. (2020). Effect of multiaxial bending-torsion loading on fracture surface parameters in high-strength steels processed by conventional and additive manufacturing. Eng. Fail. Anal..

